# Fullerol C_60_(OH)_24_ nanoparticles modulate aflatoxin B_1_ biosynthesis in *Aspergillus flavus*

**DOI:** 10.1038/s41598-018-31305-9

**Published:** 2018-08-27

**Authors:** Tihomir Kovač, Ivana Borišev, Biljana Crevar, Frane Čačić Kenjerić, Marija Kovač, Ivica Strelec, Chibundu N. Ezekiel, Michael Sulyok, Rudolf Krska, Bojan Šarkanj

**Affiliations:** 1 Josip Juraj Strossmayer University of Osijek, Faculty of Food Technology, Department of Applied Chemistry and Ecology, Franje Kuhača 20, 31000 Osijek, Croatia; 2 Center for Analytical Chemistry, Department of Agrobiotechnology (IFA-Tulln), University of Natural Resources and Life Sciences, Vienna (BOKU), Konrad-Lorenz-Str. 20, 3430 Tulln, Austria; 3 University of Novi Sad, Faculty of Sciences, Department of Chemistry, Biochemistry and Environmental protection, Trg Dositeja Obradovića 3, 21000 Novi Sad, Serbia; 4 Inspecto Ltd., Električne centrale 1, 31400 Đakovo, Croatia; 5 Department of Microbiology, Babcock University, Ilishan Remo, Ogun State Nigeria; 6 Institute for Global Food Security, School of Biological Sciences, Queens University Belfast, University Road, Belfast, BT7 1NN Northern Ireland, United Kingdom

## Abstract

The water soluble fullerene C_60_ daughter product - fullerols C_60_(OH)_24_ (FNP) possesses a great potential of modifying secondary metabolites biosynthesis. In order to clarify the extent of interaction, the impact of FNP (10, 100 and 1000 ng mL^−1^) on aflatoxin production and the available precursors of biosynthesis pathway from *Aspergillus flavus* NRRL 3251 was determined, in both the mycelia and yeast extract sucrose (YES) medium, during a 168-hour growth period at 29 °C in the dark. The FNP of 8 nm in diameter, and with a zeta potential of −33 mV affected mycelial growth at 1000 ng mL^−1^ while conidia production was slightly affected at 10 ng mL^−1^. The FNP effect on aflatoxin and it biosynthetic precursors was concentration dependent and alteration of the sterigmatocystin (ST) export from the cell was observed. Most of the monitored aflatoxin precursors, except norsolorinic acid, were detected in both mycelia and YES medium. However, observed precursor concentrations were much higher in mycelia, with exception of ST. The study shows the loss of FNP antioxidative effect after 120 hours of growth, and strong concentration dependent aflatoxigenic effect after that time. Thus, this data is relevant to guide future considerations on FNP-fungal interactions in the environments and on risk assessment.

## Introduction

Fullerols C_60_(OH)_24_ (FNP) are water soluble fullerene C_60_ daughter products with poorly defined environmental reactivity, distinctive material properties, and biological activities, which have gained great attention today. There are several available toxicity studies of FNP^1–5^; however, these studies were not carried out on fungal species. For example, Vávrová *et al*.^1^ stated that fullerols are low-toxic substances and Çavaş *et al*.^2^ reported that co-exposure with fullerol significantly reduced the cytotoxicity and genotoxicity of acetamiprid in IMR-90 cells. In addition, Milic Torres *et al*.^3^ reported the antiproliferative properties and protective effects of fullerol against doxorubicin cytotoxicity mediated by antioxidative and hydroxyl radical scavenging activity. Furthermore, Grebowski *et al*.^4^ reported that fullerol C_60_(OH)_36_ protects human erythrocyte membrane against high-energy electrons due to their reactive oxidative species (ROS) scavenging ability. Kazmierska-Grebowska *et al*.^5^, however, reported that fullerol C_60_(OH)_36_ exhibited dose-dependent effect by impairing hippocampal theta oscillations (*in vivo* and *in vitro*) and triggering epilepsy (*in vitro*) at relatively high concentrations (60 μM and 80 μM *in vitro*; 0.2 μg μL^−1^ *in vivo*) but exerting no apparent effects at lower concentrations (20 μM, 40 μM *in vitro*; 0.05 μg μL^−1^, 0.1 μg μL^−1^, 0.15 μg μL^−1^ *in vivo*).

FNP can be used as biomedical agents in chemotherapy, neurodegenerative diseases and radiobiology due to their antioxidant properties, which endows them as intensive research and promising application agents^6–8^. Increased use, environmental release, mineralization time up to 16 weeks, and limited literature about FNP – fungi interaction due to inherent properties of both mycotoxigenic fungi and FNP, are considerations that make this topic of prime importance. Fullerene C_60_ production is elevated by several tonnes per year while mycotoxigenic fungi migration is provoked by raising the environmental temperature in the face of climate change^9–11^. FNPs are mineralised in soils where the most important mycotoxin-producing saprophytic soil fungus, *Aspergillus flavus*, resides. These fungi are involved in pre-harvest and post-harvest crop colonization and contamination with aflatoxins. The mechanism of biological activity of FNP is related to their antioxidative properties, while *A*. *flavus* is sensitive to oxidative status perturbations. Literature on impact of FNP on *A*. *flavus* secondary metabolism, as a representative of mycotoxigenic fungi metabolism, is severely limited. In a bid to resolve the cause and consequences of such interaction, the impact of FNP on aflatoxin biosynthesis precursors, in both the mycelia and growth medium were determined.

## Results and Discussion

**Figure 1:**
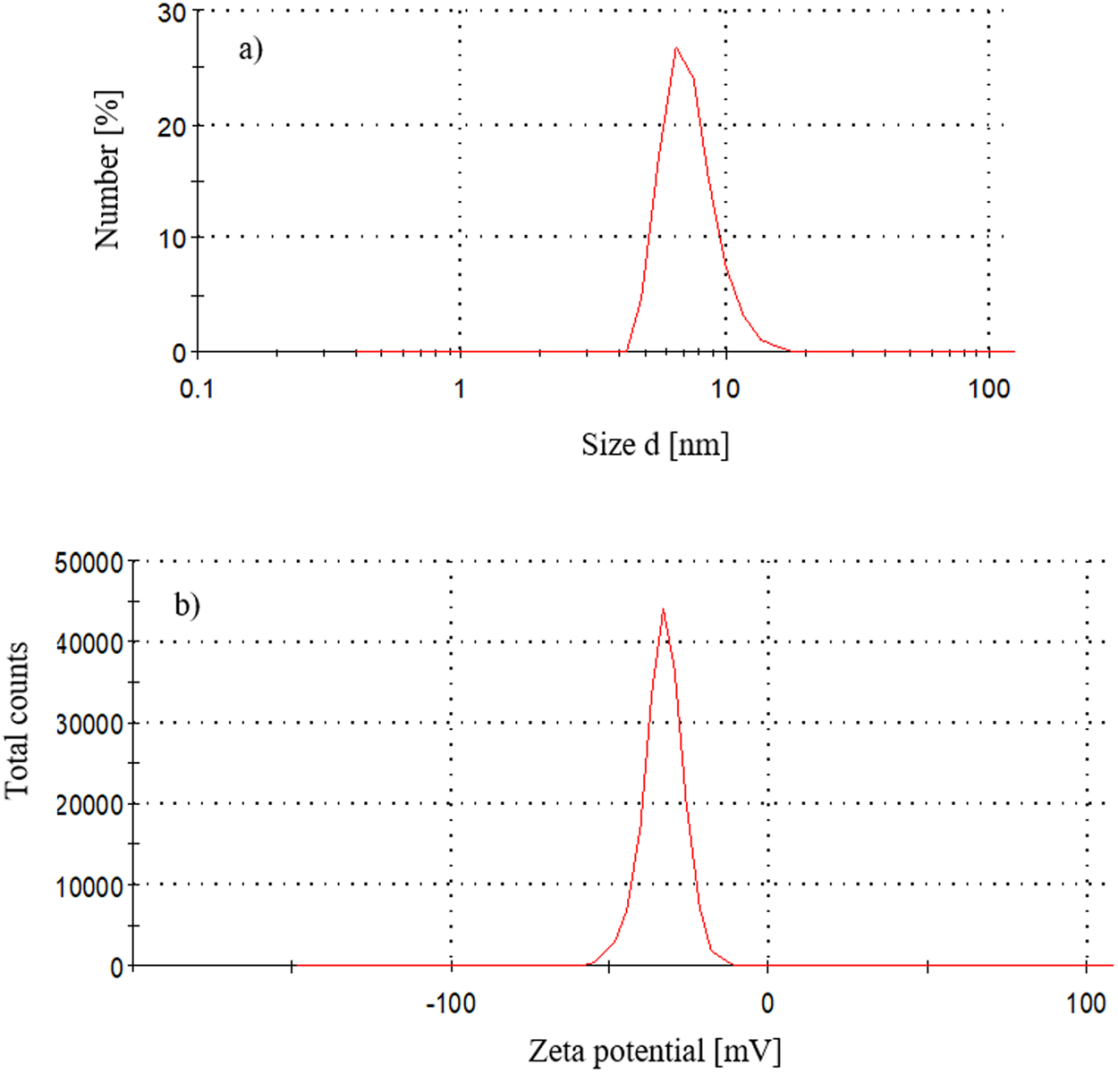
FNP aqueous solution (**a**) particle size distribution by number and (**b**) zeta potential (ζ) of FNP aqueous solution.

The particle size distribution by number of FNP in solution (10 µg mL^−1^) indicated that most of the particles had a hydrodynamic radius of 8 nm (Fig. 1a). The mean value of the zeta potential of the FNP aqueous solution was −33 mV (Fig. 1b). The data of the particle size distribution by number are in a very good agreement with the results of the particle size distribution by volume. FNP particle sizes ranged from 5 nm to 25 nm, with mean size of 8 nm as above mentioned. The DLS and zeta potential measurements of the FNP solution are consistent with previous reports^12–15^. Only particles with diameter smaller than 100 nm were present in the prepared FNP solution, this is in accordance with the EC Recommendation for the definition of the term “nanomaterial”^16^. In addition, the particle size and zeta potential data (Fig. 1), as well as theoretically calculated fullerenol C_60_(OH)_24_ molecule radius (1.1 nm), and the absence of non-agglomerated (single) molecules detection in this study, indicate the high tendency of FNPs to form agglomerates. The antimicrobial effect of FNPs is nanoparticle size-dependent^17^. Therefore, the 8 nm FNP agglomerates effect on *A*. *flavus* NRRL 3251 growth and secondary metabolism may have been due to interactions with and/or adsorption onto the fungal cell wall as proposed by Chen *et al*.^18^ and Quiao *et al*.^19^, as well as due to cell oxidative status modulation as established by Kovač *et al*.^13^.

The effect of FNP on *A*. *flavus* mycelial growth is presented in Table 1. For measurable quantity of mycelia, both in FNP-treated and non-treated samples, at least 48 hours of incubation period was needed. Altogether, there was no statistically significant effect on mycelial growth (*p* > *0*.*05*). However, it was found that *A*. *flavus* growth rate was stimulated in presence of the 1000 ng mL^−1^ of FNP. Unlike other FNP concentrations, the 10 ng mL^−1^ treated samples revealed mycelial mass reduction between 72 and 144 hours, indicating growth suppression during the logarithmic growth phase. The results of *A*. *flavus* mycelial growth stimulation under the influence of 1000 ng mL^−1^ FNP in this study are in disagreement with the reports of Aoshima *et al*.^20^ who reported the inhibition of *Malasesia furfur* and *Candida albicans* with different types of FNP at 60 to 100 times higher concentrations than those here applied. However, our report is partly in accordance with that of Gao *et al*.^21^ who reported stimulating effect of FNP on *A*. *niger* growth at concentrations higher than 10 µg mL^−1^. The disparity observed between the reports of Gao *et al*.^21^ and the present study, where the 100 ng mL^−1^ of FNP only stimulated growth at 72 and 120 hours, may have been due to the reproductive structures examined in both studies – conidia for Gao *et al*.^21^ and mycelia for the present study. The inhibition of mycelial growth at 10 ng mL^−1^ in the present study also agrees with the reduction of biomass yield of *A*. *flavus*, *A*. *parasiticus* and *A*. *ochraceus* by single low dose of FNP (5.2 ng mL^−1^) during a 120 h growth study^22^.

**Table 1:** Influence of fullerenol C_60_(OH)_24_ nanoparticles (FNP) on *A*. *flavus* NRRL 3251 mycelial growth (expressed as gram of dry weight (g.d.w.) per 50 mL) in YES medium incubated over a 168-hour period at 29 °C.

Time/hr	Mycelia weight [g(d.w.) 50 mL^−1^]			
	Control	Tested concentration [ng mL^−1^]		
		10	100	1000
48	24.94 ± 2.03^a^	22.41 ± 3.63^ax^	23.26 ± 5.90^ax^	31.04 ± 4.12^ax^
72	41.80 ± 3.22^a^	48.03 ± 2.87^ax^	58.16 ± 1.95^ax^	48.30 ± 1.64^ax^
96	43.81 ± 5.06^a^	42.89 ± 5.93^ax^	38.92 ± 7.93^ax^	47.72 ± 3.88^ax^
120	54.90 ± 1.79^a^	33.11 ± 5.41^ax^	60.72 ± 2.43^ax^	70.53 ± 8.66^ayx^
144	60.13 ± 2.39^a^	66.64 ± 4.26^ax^	48.44 ± 3.00^ax^	54.02 ± 6.06^ax^
168	58.80 ± 3.22^a^	53.52 ± 1.31^ax^	52.95 ± 1.01^ax^	63.44 ± 8.72^ax^

Data represent the mean ± SEM from three separate experiments. Values in the same row marked with superscripts alphabets (a and b) represent differences between control samples and samples treated with different concentrations of nC_60_, while values in the same row marked with superscripts alphabets (x and y) represent differences between different nC_60_ concentrations. Significance level was set at 0.05.

**Figure 2:**
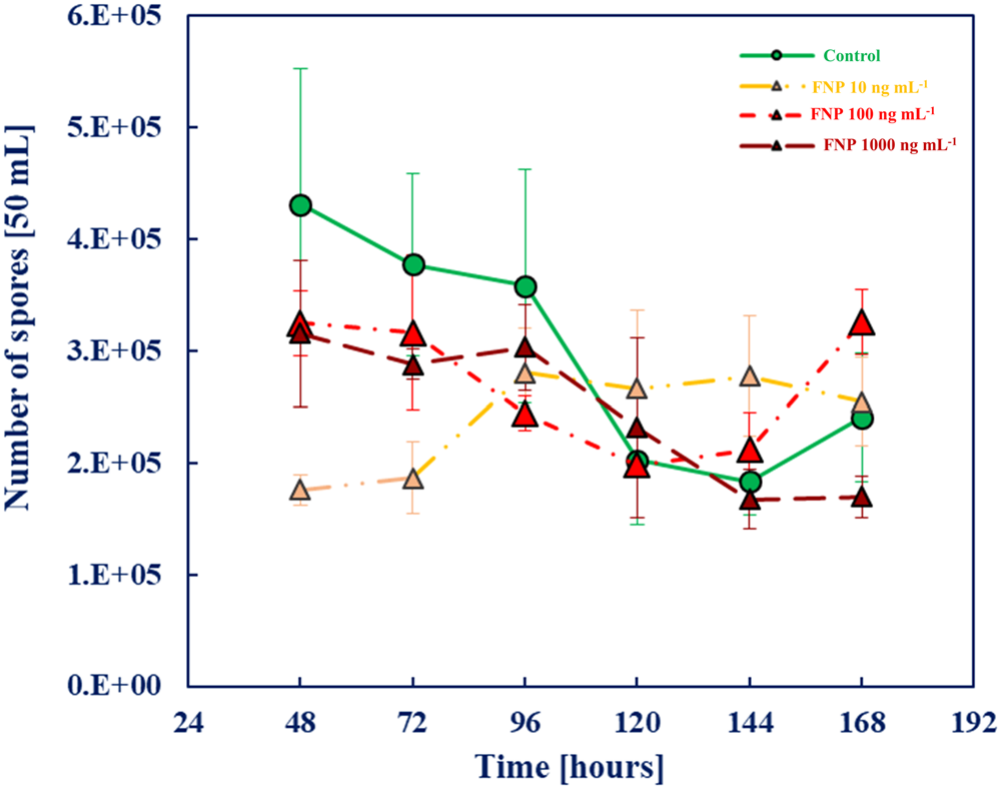
Influence of fullerenol C_60_(OH)_24_ nanoparticles (FNP) on *A*. *flavus* spore production during growth in YES medium for 168 h at 29 °C. Data represent the mean ± SEM from three separate experiments.

The effect of FNP on *A*. *flavus* conidia production ability is presented in Fig. 2. Collectively, FNP does not affect the conidia production ability of the fungus at statistically significant rate until 96 hours of growth. This is the first available data on conidia production under FNP influence. Furthermore, there is limited data and a knowledge gap regarding the FNP and mycotoxigenic fungi interaction, with the exception of reports of Unković *et al*.^22^ and Kovač *et al*.^13,23^. The relation between oxidative and/or drought stress, conidia production and aflatoxin biosynthesis are well documented^24–27^, and a change in the oxidative status of aflatoxigenic fungi due to FNP aflatoxin B_1_ (AFB_1_) production modulation was reported by Kovač *et al*.^9,13^. The results presented in Fig. 2 therefore probably reflects the impact of FNP on *A*. flavus cell oxidative status (up to the 96^th^ hour of growth) as reported by Kovač *et al*.^13^, which brings the potential for reflection on the aflatoxin production.

**Figure 3:**
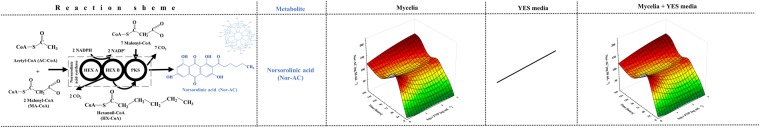
Influence of fullerenol C_60_(OH)_24_ nanoparticles (FNP) on initial steps of aflatoxin biosynthesis in peroxisomes during *A*. *flavus* growth in YES medium for 168 h at 29 °C. Data represent the mean ± SEM from three separate experiments.

**Figure 4:**
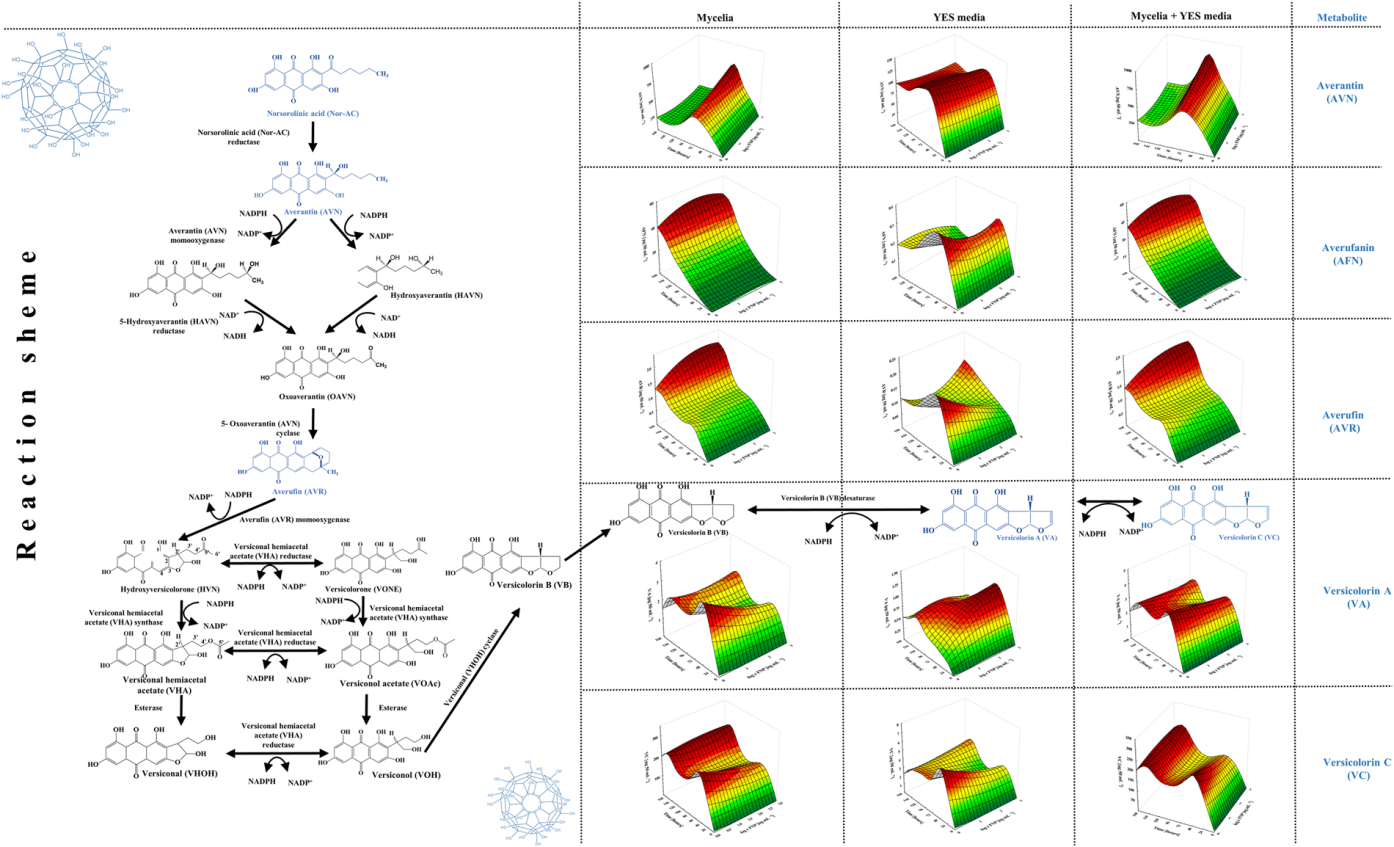
Influence of fullerenol C_60_(OH)_24_ nanoparticles (FNP) on aflatoxin biosynthesis pathway – conversion of norsorolinic acid to versicolorin B during *A*. *flavus* growth in YES medium for 168 h at 29 °C. Data represent the mean ± SEM from three separate experiments.

**Figure 5:**
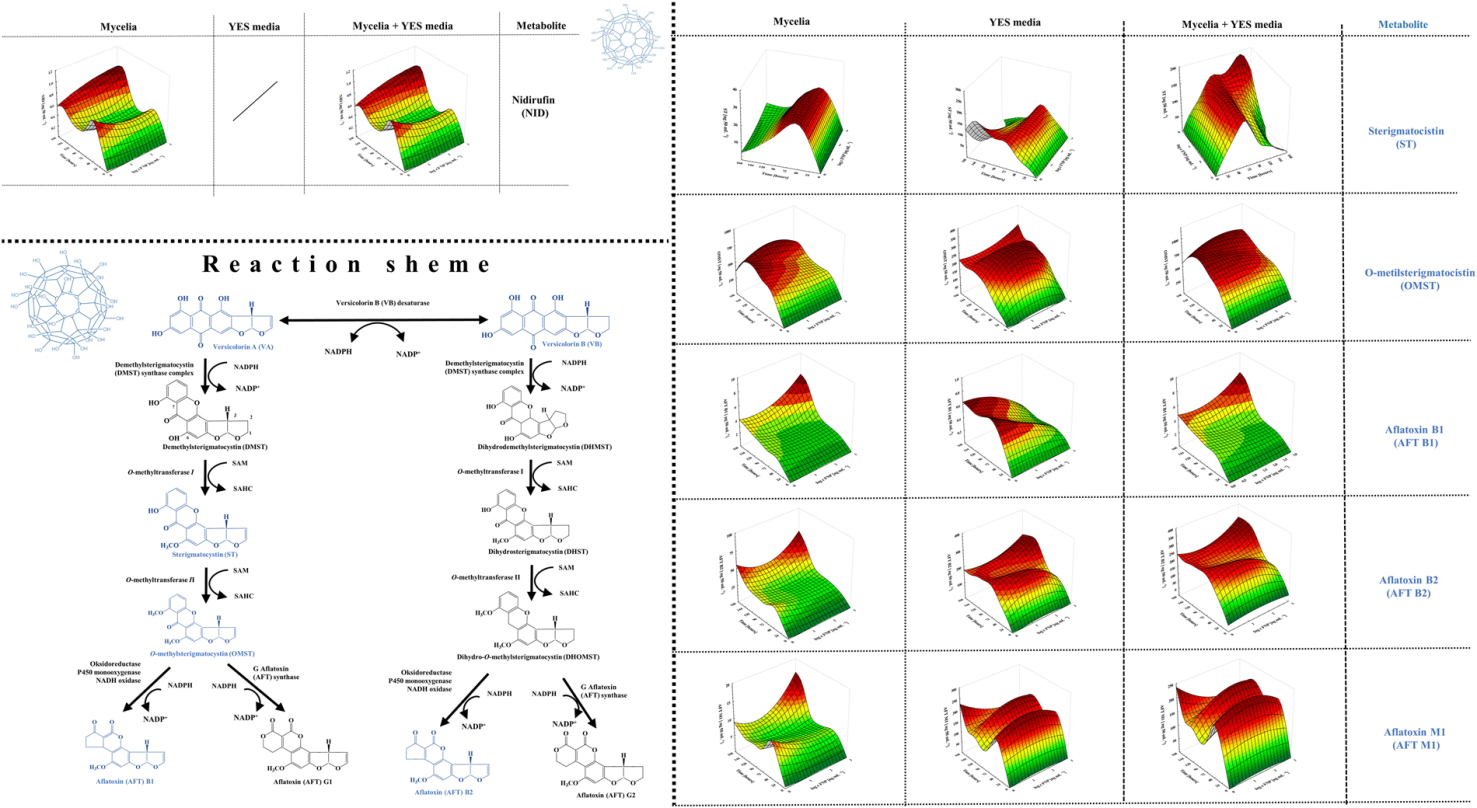
Influence of fullerenol C_60_(OH)_24_ nanoparticles (FNP) on aflatoxin biosynthesis pathway – conversion of versicolorin B to aflatoxin during *A*. *flavus* growth in YES medium for 168 h at 29 °C. Data represent the mean ± SEM from three separate experiments.

Therefore, if the antiaflatoxigenic effect is expected, according to Holmes *et al*.^28^ there is obvious assumption that FNP could alter signalling inputs perceived by fungus cell, interfere with signal transduction and gene expression regulatory networks upstream of AFB_1_ biosynthesis or block a biosynthetic enzyme activity. Due to such assumption, the available aflatoxin precursors of the biosynthetic pathway (Figs 3–5) of *A*. *flavus* NRRL 3251 secondary metabolism in FNP presence were determined, in both the mycelia and growth medium. In addition, listing of aflatoxin precursors in the group of “emerging toxins”^29^ is additional reason to determine their concentrations in the presence of FNP. Such data are necessary for risk assessment, i.e. potential future legislation regarding the FNP production and release.

The aflatoxin biosynthetic pathway is proposed to originate in peroxisomes (Fig. 3), continues with multiple fusion events in vesicles and ends with formation of aflatoxisomes – the large bodies containing aflatoxins and their precursors (Figs 4 and 5)^27,30,31^.

The initial reactions of this energy expensive biosynthetic pathway (1 AC-CoA, 9 MA-CoA, <10 NADPH, 2 SAM, 9 ATP) begins in peroxisomes by norsorolinic acid (Nor-AC) synthesis^31–33^. The effect of FNP on Nor-AC concentration is shown in Fig. 3. Nor-AC was detected only in mycelial samples; both in FNP treated and control samples. The most probable reason for lack of Nor-AC in growth medium is its rapid conversion in further steps of the pathway. Such results for Nor-AC are in accordance with the model for compartmentalization, translocation, and aflatoxisome biogenesis during biosynthesis of aflatoxin in Aspergilli as proposed by Rose *et al*.^34^. However, FNP at all tested concentrations caused an average decrease of 67% of Nor-AC in mycelia until 72 hours of growth in comparison to the control growth. The Nor-AC concentration increased after 72 hours of growth under FNP influence while growth concentration remained constant in control samples. After 144 hours of growth, FNP caused increase of Nor-AC biosynthesis up to 42% at 100 ng mL^−1^ while at 10 and 1000 ng mL^−1^ FNP increase of Nor-AC production was at 25% rate (Fig. 3). This implies possible involvement of FNP in early stages of aflatoxin biosynthesis.

The next two steps of the aflatoxin biosynthetic pathway occur in the cytosol (Figs 4 and 5). The pathway enzymes and precursors are translocated into the cytosol via vesicles that originate from budding of peroxisomes and mitochondria, and they fuse with trafficking and secretory vesicles, altogether forming aflatoxisomes - large bodies containing substantial aflatoxin concentrations, pathway enzymes and aflatoxin precursors^31,34,35^.

At first, Nor-AC is converted to averantin (AVN) then to averufin (AVR), with averufanin (AFN) as intermediate. After multiple reactions, AVR is converted to versicolorins B (VB), A (VA) and C (VC)^36^. The effects of FNP on concentrations of the hydroxyantraquinones AVN, AFN, AVR, VA and VC are shown in Fig. 4. VB is synthesised and converted to aflatoxin by multiple enzyme reactions (Fig. 5). At first, sterigmatocystin (ST) and *O*-methylsterigmatocystin (OMST) are formed, and later the aflatoxins B_1_ and B_2_ at the end of the biosynthetic pathway. All of mentioned aflatoxin precursors (Figs 4 and 5) were biosynthesised during the entire incubation period, and were present in mycelia and also released into the growth medium. The concentrations of AVN, AVR, AFN, VA and VC in the mycelia were on average 2.3–3.6, 7.9–14.4, 105–255, 2.6–6.0 and 61.2–80.1 times higher than in the medium, respectively. The sum of AVN concentrations (mycelia and medium) was not significantly affected by FNP, except at 100 and 1000 ng mL^−1^ FNP for 72 hours of growth where an increase of 95 and 72%, respectively, were recorded. In the case of AFN, an increase in the sum of mycelia and medium concentrations was recorded after 144 hours of growth for 10 ng mL^−1^ (32%) and 100 ng mL^−1^ (up to 53%) FNP. The sum of AVR concentrations in mycelia and medium, however, showed about 23% decrease until 72 hours of growth, while an increase in AVR concentration was observed for all FNP concentrations after 144 hours, especially at 100 ng mL^−1^ (up to 80%). Furthermore, nidirufin (NID), C-2′hydroxyl derivative of AVN^37^, was observed only in mycelial samples. Its concentrations decreased by 51% under FNP influence until 72 hours and increased by as much as 58% after 144 hours of growth (Fig. 5). NID is one of the by-products in the transformation process of AVN to VA^36^. VA is the result of multiple enzyme transformation of AVR to VB, which can be further transformed to VC. The sum of VA concentrations decreased under FNP presence at 72 hours of growth. VC is a “side shunt metabolite” of the aflatoxin biosynthesis pathway and is regarded as non-toxic^36^. The sum of its concentrations in mycelia and medium decreased under FNP presence from 48 to 144 hours of growth (Fig. 4).

The most toxic of the AFB_1_ precursors, which has been assigned to group 2 (possibly carcinogenic to humans) by the International Agency for Research on Cancer (IARC 2012), is ST^38^. ST is one of the few metabolites found in higher concentrations in medium than in mycelia, indicating possible active transport out of the fungal cell. However, the concentrations in the sum of ST (mycelia and medium) showed 50% increase until 72 hours, and the 95% decrease after 96 hours of growth, all in the presence of FNP. This implies an early export of ST from the cell until 72 hours of growth due to FNP. The results recorded for ST are also in accordance with the observed sum of concentrations of OMST. The sum of AFB_1_ concentrations in mycelia and medium decreased under FNP presence until 120 hours of growth at average rate of 58%, but increased afterwards in a concentration dependent manner at an average rate of 22% until 168 hours of growth, where the highest FNP concentration applied showed the strongest aflatoxigenic effect. However, a higher FNP effect occurred in the case of AFB_2_, where a concentration dependent aflatoxigenic effect leading to a 50% average concentration increase at highest applied FNP concentration after 120 hours of growth was observed. In addition, AFM_1_ was detected in both mycelia and medium, and the sum of the concentrations revealed the strongest aflatoxigenic effect at an average rate of 93% until 144 hours of growth at the lowest FNP concentration (Fig. 5). AFM_1_ is primarily found in animal tissues and fluids (milk and urine) as a metabolic product of AFB_1_^39^ but is also found as a product of secondary metabolism of *Aspergillus* species^40–43^. There was no observed statistically significant difference between the ratio of aflatoxins B_1_ and B_2_ for the applied FNP concentrations. In general the ratio between AFB_1_ to AFB_2_ was in accordance with literature, on average 93 ± 4% of AFB_1_, and 7 ± 4% of AFB_2_ was produced^44^. Furthermore, the correlation between conidia production and AFB_1_ production was statistically significant for control samples (r = 0.85838; p = 0.0159) and FNP treated samples at 10 ng mL^−1^ (r = 0.86229; p = 0.0152), 100 ng mL^−1^ (r = 0.90389; p = 0.0189) and 1000 ng mL^−1^ (r = 0.22522; p = 0.00011).

This work presents updated information on the most detailed and available study on FNP effect on growth and aflatoxin producing ability of *A*. *flavus*^13,23^. The novelty of this study is in the determination of every available aflatoxin precursor in the biosynthetic pathway of *A*. *flavus* NRRL 3251 secondary metabolism. Previously Kovač *et al*. (2017)^13^ demonstrated the antiaflatoxigenic effect of FNP in YES medium and the published data did not include mycelial AFB_1_ content; thus, the hypothesis that mycelial mycotoxin content could be different was formulated. The main reason for this assumption is that the antioxidative potential of FNP is limited with time such that the proven ability of FNP to modulate oxidative stress within the fungal cell, even at relatively low doses, disappear after some time (Fig. 5). Sequel to the initial antioxidative activity of FNP, the AFB_1_ production increases due to oxidative stress modulation (i.e. higher oxidative stress levels), and the increased oxidative stress is one that is known to trigger AFB_1_ biosynthesis^45^. The data in the present study expatiates the previous work by Kovač *et al*.^13^, which was on a preliminary investigation into the modulation of mycotoxin production by FNP. Furthermore, this present study addresses issues of study design, i.e. importance of metabolite determination in both mycelial and media, FNP alteration ability into aflatoxin biosynthetic pathway precursors export, as well as AFB_1_ accumulation within the cells.

## Conclusion

This study has shown that FNP modulates aflatoxin production by affecting the biosynthetic pathway of metabolites reported herein. When considering environmental risk estimation, the sum of mycotoxin concentrations from mycelia and growth medium should be considered to determine FNP impact on toxigenic fungi. Nanoparticles as an environmental threat are concentration-dependent, and trends of FNP concentration increase are expected in the future due to increased production, usage, release, and time-lag in the mineralisation processes in the environment. Thus, the environmentally occurring FNP levels are capable of posing threats to the toxigenic potentials of mycotoxigenic moulds (e.g. *A*. *flavus*), especially in the face of climate change. In this study, FNP exerted a concentration-dependent effect on the aflatoxin biosynthesis pathway and an alteration in ST export from the cell occurred. Prior to 120 hours of growth of *A*. *flavus*, FNP exerted antioxidative potential which vanished afterwards to cause strong and concentration-dependent rise in biosynthesis of aflatoxins. Consequently, such negative prospects of FNP application evoke thoughts on investigating photosensitised FNP action on *A*. *flavus* and other mycotoxigenic fungal species as an option of controlling aflatoxigenicity in the future.

## Material and Methods

### Chemicals

Yeast extract, potato dextrose agar and sucrose were purchased from Biolife (Italy) while aflatoxin standard mix (B_1_, G_1_, B_2_, G_2_) was purchased from Biopure (Austria). Acetonitrile and methanol (both HPLC grade) were obtained from Merck (Germany). Ammonium acetate and glacial acetic acid (p.a.) were purchased from Sigma Aldrich (Vienna, Austria). A Purelab Ultra system (ELGA LabWater, Celle, Germany) was used for ultrapure water preparation. Standards of *A*. *flavus* metabolites were purchased from Various research groups or from the following commercial sources: Romer Labs®Inc.(Tulln, Austria), Sigma–Aldrich (Vienna, Austria), Iris Biotech GmbH (Marktredwitz, Germany), Axxora Europe (Lausanne, Switzerland) And LGC Promochem GmbH (Wesel, Germany) and prepared as described by Malachova *et al*.^46^.

### Fullerol C_60_(OH)_24_ synthesis, preparation and characterisation of nanoparticles solution

Fullerol C_60_(OH)_24_ was synthesised and nanoparticle solution prepared in ultrapure water as previously described by Mirkov *et al*.^47^. Dynamic light scattering (DLS) technique was used for the determination of hydrodynamic size, and electrophoretic light scattering (ELS) for the measurements of the surface charge (zeta potential (ζ)) of analysed samples. The measurements were conducted on a Zetasizer Nano ZS instrument (Malvern Instruments Inc., UK). All DLS analyses were performed in triplicates at 633 nm wavelength and a measurement angle of 173^o^ (*backscatter detection*), in an aqueous solution at room temperature. Zeta potential (ζ) measurements were performed in duplicate.

### *Aspergillus flavus* growth and spore count determination

The preparation of conidia suspension of *A*. *flavus* NRRL 3251, inoculation, as well as mycelial growth in aflatoxin-inducing YES medium were conducted in the dark at 29 °C which favours aflatoxin production as previously described (Kovač *et al*.)^13^. The inoculated flasks were incubated on a rotary shaker (KS 260 basic, IKA, Germany) at 200 rpm for 168 hours in the presence of environmentally plausible C_60_(OH)_24_ concentrations (0, 10, 100 and 1000 ng mL^−1^). Samples of medium and mycelia were collected from the flasks every 24 hours from the 48^th^ to 168^th^ hour of incubation. Mycelia were separated from the media by filtration, mycelia precooled and stored in 2 mL tubes at −80 °C for at least 24 hours until lyophilisation (Christ, Alpha 1–4 LD, Germany). Drying conditions were as follows: freezing temperature −55 °C; temperature of sublimation −35 to 0 °C; vacuum level 0.220 mbar. The temperature of isothermal desorption varied from 0 to 22 °C under the vacuum of 0.060 mbar. Freeze-drying lasted until the constant mass of mycelia was obtained, which was about 5 h. In addition, a part of mycelia prior to −80 °C storage and lyophilisation was dried until constant mass (24 hr at 105 °C) in order to determine dry mycelial weight.

The conidia count in YES medium after separation was performed as previously described^13^.

### Estimation of *A*. *flavus* metabolites in mycelia and culture media

For the estimation of *A*. *flavus* metabolites in mycelia and culture medium, the multi-analyte “dilute and shoot” LC-MS/MS method was used as previously described^46^.

The 125 mg of the lyophilised mycelia were mixed with 1 mL of extraction solvent (acetonitrile/water/acetic acid 79:20:1, v/v/v) and extracted for 90 min at GLF 3017 rotary shaker (GLF, Germany). Extracts were transferred into glass vials and two-fold diluted with dilution solvent (acetonitrile/water/acetic acid 20:79:1, v/v/v). Vial contents were vigorously mixed and 5 µL was injected directly into LC-MS/MS system. The metabolites in YES medium were estimated by following a ten-fold dilution with dilution solvent in glass vials without any pre-treatment, as well. The screening and detection of metabolites was the same as described by Malachová *et al*.^46^, in brief the QTrap 5500 MS/MS detector (Applied Biosystems, Foster City, CA) equipped with TurboV electrospray ionization (ESI) source, and Agilent 1290 binary UHPLC system (Agilent Technologies, Waldbronn, Germany) was used. For the separation of the metabolites the Gemini® C18 column (150 × 4.6 mm i.d., 5 µm particle size) was combined with the C18 security guard pre-column (4 × 3 mm i.d.) (Phenomenex, Torrance, CA, US). The eluents were freshly prepared, the gradient and the flow rate were followed exactly as described by Malachová *et al*.^46^. The Scheduled selected reaction monitoring (sSRM) mode was used, and two runs per sample were used (each for one mode). The detection window was set to ±27 s in positive and ±42 s in negative mode due to number of monitored metabolites. The ESI source parameters were followed exactly as described by Malachová *et al*.^46^. Two sSRM transitions were monitored per metabolite (quantifier, and qualifier), and according to the validation guidelines, the ratio between two transitions were used as additional identity conformation point.

### Statistical analysis

Data presented in this paper are expressed as the mean value ± SEM from three separate experiments. The pooled datasets were checked for distribution normality by Shapiro-Wilk test and compared by nonparametric statistics methods (Friedman ANOVA and Kendall coefficient of concordance; Kruskal-Wallis ANOVA). The programme package Statistica 12.0 (Dell, 2015) was used and differences were considered significant when the *p* value was < 0.05.
